# Policies and strategies to control antimicrobial resistance in livestock production: A comparative analysis of national action plans in European Union Member States^☆^

**DOI:** 10.1016/j.healthpol.2024.105238

**Published:** 2025-02

**Authors:** Caetano Luiz Beber, Maurizio Aragrande, Massimo Canali

**Affiliations:** a JRC, Joint Research Centre, European Commission, Edificio EXPO, c/Inca Garcilaso 3, E-41092 Seville, Spain; b Department of Agricultural and Food Sciences, University of Bologna, Via G. Fanin 50, Bologna 40127, Italy

**Keywords:** Livestock production antimicrobial use policies, Livestock production antimicrobial resistance policies, Animal health economics, One health

## Abstract

Policy strategies targeting imprudent antimicrobial use (AMU) in livestock farming have been established at the global and country levels, recognising the risks associated with antimicrobial resistance (AMR). This study evaluates the strategies addressing AMU and AMR in animal farms and the food supply chain in EU Member States using a multimethod approach. Our aim is to contribute to the debates surrounding the goals set by the EU Commission and the ‘Strategic framework for collaboration on antimicrobial resistance: Together for One Health’. We first review the policies, strategies and specific legislation in the European Union (EU) and Member States about AMU/AMR in livestock production. We then evaluate the national action plans for AMU reduction in the EU using the progressive management pathway tool from the FAO. Finally, we assess the measures that affect AMU reduction by applying a system generalised method of moments to a 8-year panel of the same countries. According to our results, efforts to reduce AMU could be focused on controlling excessive AMU in the pig sector. Further veterinary training on AMU/AMR and improvements in the performance of the veterinary sector, as well as strengthening the development of multisector and One Health collaboration and coordination, can also contribute to achieving better standards in AMU reduction in the livestock sector and, consequently, for AMR control.

## Highlights

• EU-MS show varied progress in NAPs for AMU and AMR in livestock.
• Strong governance and One Health collaboration are key to reducing antimicrobial consumption.
• Enhanced veterinary education on AMU/AMR leads to significant reductions in antimicrobial use.
• Monitoring systems for AMU and AMR are crucial for informed policy-making/intervention.
• Policies must address past AMU patterns and focus on specific livestock sectors.

## 1 Background

Antimicrobial resistance (AMR) is a pressing global concern that poses significant threats to both public health and food security. The misuse and overuse of antimicrobials (AMs) in various regions are major contributing factors to the emergence and spread of AMR [1]. While AMs are essential for human and animal health, it has been projected that the majority of future antimicrobial use (AMU) growth will occur in animal production. In fact, today, the use of AMs is already highest in animal production [2,3]. Managing AMR in the livestock sector is challenging given the diverse contexts in which it occurs.

The complexity of the AMR issue is evident when examining healthcare systems globally, as well as within EU Member States that are at different stages of reform and transition [4]. Despite a few good examples, there have been insufficient efforts to regulate the prudent use of AMs or provide comprehensive guidance on their use. There is limited access to sensitivity testing to guide the use of AMs, particularly in veterinary settings [5]. In most countries, there is a lack of education on the prudent use of AMs among medical, veterinary and other health-related professionals, leading to overprescribing and misuse [6]. Furthermore, the shortage of new effective drugs against bacterial infections presents an additional challenge in the face of the increasing spread of resistant pathogens [7,8]. The increasing interconnectedness between countries and the globalisation of trade and travel further heighten the risk of importing bacteria or genes that undermine effective treatment or the prevention of bacterial infections. This emphasises the need for international standards, governance and data sharing [9].

To combat the health risks associated with the inappropriate use of AMs in livestock farming, policy strategies have been formulated at the international, European and national levels. These strategies acknowledge the increasing burden of AMR and recognise the role of AMU in food-producing animals in contributing to this problem. International organisations, namely FAO, WOAH, WHO and UNEP have collaborated through a quadripartite agreement to develop global action plans on AMR. The European Union (EU) also promoted a coordinated joint strategy among its Member States to minimise the burden of AMR. In 2011, the European Commission delivered an action plan against the rising threats from AMR [10]. The initiative was relaunched in 2017 through the EU One Health action plan against AMR [11], endorsing the WHO global action plan issued in 2015, and the Member States committed themselves to develop national action plans (NAPs). In 2020, the European Commission launched the farm-to-fork strategy, setting the target of a 50 % reduction (from 2018 values) in the overall EU sales of AMs for farmed animals by 2030 [12], although without clear indications to achieve the target [13,14].

Addressing the AMR problem extends to food systems, with several instances where formal arrangements, including regulations, are being imposed by private entities. For example, in Denmark's pig sector, concerted efforts between public and private sectors have been fruitful in meeting political targets and driving change to mitigate AMR. This collaborative approach has succeeded in co-creating incentives that have made a significant impact [15]. Sweden has demonstrated a producer-led initiative through the establishment of the Swedish Farmer's Disease Control Program in 1995. This program promotes health checks of imported animals and sets additional voluntary import requirements. Moreover, the collaboration between various stakeholders, including authorities, farmers, trade organizations, and food companies, has significantly reduced the prevalence of resistant bacteria such as ESBL-forming E-coli in poultry [16,17]. In the Netherlands, a public-private partnership gave rise to the Netherlands Veterinary Medicines Authority (SDa). This partnership involves the government, the Royal Dutch Veterinary Association, and livestock industries. Private quality assurance systems known as IKB have implemented regulations that greatly limit the use of certain antibiotics in farm animals. In 2011, the PVE, a public-private organization with legislative power, introduced the Farm Health Plan and Farm Treatment Plan. These initiatives require the registration of all prescribed and delivered antimicrobials on farms. They have been integrated lately into existing IKB systems across various livestock sectors [18].

Consumer behavior is also shifting. Influenced by retailers capitalizing on product differentiation opportunities, an increasing number of consumers are purchasing antibiotic-free livestock products, particularly fresh products of animal origin [19,20]. This change in purchasing habits, driven primarily downstream in agri-food supply chains, further emphasizes the multifaceted approach necessary to effectively combat AMR.

A deep understanding of the different policy measures, strategies and goals within the various country contexts is necessary to the strengthening of global AMR containment. This understanding can shed light on and provide evidence to tackle the complexities of public health systems and social challenges associated with AMR worldwide. It can also help identify which measures are better suited and more effective in different situations, guiding future policies and interventions. Therefore, this study aims to review and assess the current policies and strategies of EU Member States in their fight against AMR and for the reduction of AMU in livestock production in order to strengthen the One Health actions and the goals of the EU. It is structured as follows. Section 2 describes the multimethod approach. Section 3 details the historical evolution of the European policy framework about AMU and AMR. Section 4 provides statistical evidence on which measures bring about a lower AMU and thus make a direct contribution to achieving the EU Member States’ individual targets. For this analysis, we use 8 years’ panel data from 27 EU Member States using a system generalised method of moments (SYS-GMM) model. Section 5 discusses the results and the report closes with the concluding remarks on Section 6.

## 2 Methods

This study uses a multimethod approach to address the AMR/AMU issue in the EU from different perspectives. In Section 3 we provide an overview of the legislation, policies and strategies in the EU and EU Member States on AMR/AMU at the livestock sector. To gather a first round of information and documents about such policies, a questionnaire was sent to partners in the H2020-Roadmap (Rethinking of antimicrobial decision-systems in the management of animal production) project, containing questions about the existence and type of legislation in their respective countries. Documents and translations of them, when available, were also requested. These were complemented by a second round of data collection based on a review of policies in the EU and other partners not represented in the project. With these data and information, we created a timeline of policies and legislation to understand where and when this issue was first considered a relevant problem worth being addressed by policymakers. We also classified each of these actions according to FAO categories from the AMR policy review [22], which provides a holistic overview of the policies and strategies within a progressive pathway of improvement. The four policy domains can be described as follows.

• **Awareness.** Improve awareness of AMR and related threats. Raise awareness and understanding of AMR through effective communication, education and training.
• **Evidence.** Develop capacity for surveillance and monitoring of AMR and AMU in food and agriculture. Understand the extent of AMU and AMR in the food and agriculture sectors through data generation as a basis for driving action.
• **Good practices.** Promote good practices in food and agriculture systems and the prudent use of AMs. Develop and support practical measures to be taken in the food and agriculture sectors to minimise the need for AMs, and optimise the use of AMs, to minimise or prevent the spread of AMR:
  ○ responsible use – reducing public demand and supply by prescribers and dispensers,
  ○ infection prevention and control to reduce the overall need for AMs.
• **Governance.** Strengthen governance related to AMU and AMR in food and agriculture. Enhance political commitment, improve policy and ensure that there is a relevant regulatory framework to provide the capacity and resources to combat AMR. Develop and implement a multisectoral NAP on AMR.

In Section 4 we employed a panel data covering 27 countries over 8 years. This data structure allows dynamic estimations to be performed, considering the heterogeneity between countries without dynamic bias due to data aggregation [23]. Following recent studies [[24], [25], [26], [27]], we consider a dynamic model that includes lagged dependent variables as instruments, accounting for autocorrelation while allowing us to obtain consistent estimates of the other parameters [28]. We therefore applied a SYS-GMM, which combines a set of equations in levels using lagged differences as instruments and provides more efficient estimations [29].

The objective is to explain the veterinary consumption of AMs (in mg per population correction unit (PCU)) from the European Surveillance of Veterinary Antimicrobial Consumption (ESVAC) database [30]. Thus, as a first step, 8 years’ panel data from 27 EU Member States was constructed considering all available years from ESVAC (2015 to 2022). We looked to explain countries’ veterinary AM consumption with variables representing their policies and strategies related to awareness, evidence, practices and governance of AMU and AMR. As regressors, we considered the variables from TrACSS where countries self-assessed their own policies and strategies.

For this, a scoring system for variable construction was adopted for 2016/2017 to 2023 (see supplementary material). We retained only variables for which data were available in the 8-year period. Thus, seven variables, one for awareness and two each for evidence, practices and governance, were included in the model (Table 1 and supplementary material TrACSS questions). We also included control variables that represent the proportion of pigs and poultry (in PCU) in the countries’ livestock (accounting for cattle, poultry and pigs) using FAOSTAT data [31]. Pigs and poultry are reported as being the species for which the largest amount of AMs is used during the production process in various countries [32,33]. In the TrACSS, the countries’ responses range from A to E , or ‘Yes’ or ‘No’, depending on the question. To compare countries, we assigned scores on a scale from 1 to 5 (Where A = 1 and E = 5, No = 0, and Yes = 1), based on the responses.

**Table 1:** NAP variables included in the analysis.

CATEGORY	Variable	Description according to TrACSS
Awareness	VET_edu	Training and professional education on AMR in the veterinary sector
Evidence	AMU_mon	National plan or system in place for monitoring sales/use of antimicrobials in animals (also crops in 2016)
	AMR_surv	National surveillance system for antimicrobial resistance in live terrestrial animals
Good practices	VET_serv	Progress with strengthening veterinary services
	BIOSEC_pract	Biosecurity and good animal husbandry practices to reduce the use of antimicrobials and minimize development and transmission of AMR in terrestrial animal production
Governance	OneHealth	Multisector and One Health collaboration/coordination
	NAP_prog	Country progress with development of a National Action Plan on AMR

The final model specification is as follows:

(1) $$MgPCU_{i,t}=\alpha +\beta MgPCU_{i,t-1}+\gamma X_{i,t}+\delta Z_{i,t}+\theta_{t}+v_{i}+\varepsilon_{it},$$

here $MgPCU_{i,t}$ is the country's consumption of AMs measured in sales of AMs in Mg/PCU, $MgPCU_{i,t\;-\;1}$ is the consumption in the preceding year, $X_{i,t}$ is a vector of variables representing the policies and strategies of countries related to AMU and AMR, $Z_{i,t}$ represents a control variable for the proportion of pigs in PCU related to cattle and poultry, $\theta_{t}$ are dummy variables for each year, $v_{i}$ is the unobserved country-specific effect and $\varepsilon_{it}$is the random disturbance term.

## 3 The European Union policy framework and strategies

The EU Member States provide several examples of recent action at the national and regional levels in the fight against AMR according to the One Health approach, focusing on measures of awareness, monitoring, good practices and governance. Concerns about continuous, low-dose use of AMs in animal feed, without any veterinary prescription, for the purpose of improving growth and feed conversion in production animals (AGPs) were raised in several European countries soon after the approval of such use back in the early 1950s [34]. These concerns led to a report issued by the Swann Committee [35], which was established by the UK government in 1969, calling for restricted use of AGP due to the risk of resistance development.

The Swann Committee report argued that AMs in livestock, particularly in subtherapeutic doses, may imply certain hazards to human and animal health, and only AMs that have little or no application as therapeutic agents should be used as AGPs. The report's conclusions contributed to the first limitations on the use of AMs as AGPs in the European Community on additives in feeding-stuffs (see Table 2). This was the first legislative harmonisation among the European Community's countries on medicated animal feed [36].

**Table 2:** Timeline of EU legislation/action on AMs in livestock production.

Timeline	Legislation/action	
Before 1990	1950: started the use of AMs in livestock production	FAO classification
	Council Directive 70/524/EEC: limitations on the use of antibiotics important for therapeutic use in humans and animals (penicillin, streptomycin and tetracyclines) as AGPs	Practices (ban)
1990s	Council Directive 90/167/EEC laying down the conditions governing the preparation, placing on the market and use of medicated feedingstuffs in the Community	Practices (regulation)
	Council Directive 92/117/EEC: measures for protection against specified zoonoses and specified zoonotic agents in animals and products of animal origin in order to prevent outbreaks of food-borne infections and intoxications	Practices (regulation/control)
	Council Directive 96/23/EC: measures to monitor certain substances and residues thereof in live animals and animal products	Evidence
	Commission Directive 97/6/EC of 30 January 1997: ban on use of avoparcin	Practices (ban)
	Since 1998, EARS-Net, now run by ECDC, has collected information from all EU Member States	Evidence
	Council Resolution of 8 June 1999 on antibiotic resistance ‘A strategy against the microbial threat’	Governance
2001	Communication from the Commission on a Community strategy against antimicrobial resistance: recognises the importance of fighting AMR. First policy instrument to address AMR at the European level in four areas: surveillance, prevention and control, research and product development, and international cooperation COM(2001) 333 final	Governance
	Directive 2001/82/EC on the Community code relating to veterinary medicinal products	Practices (regulation)
2003	Regulation (EC) No 1831/2003: banned the use of antibiotics as growth promoters on European Union farms from 2006	Practices (ban)
	Directive 2003/99/EC of the European Parliament and of the Council of 17 November 2003 on the monitoring of zoonoses and zoonotic agents. Monitoring Antimicrobial Resistance in the EU (EFSA)	Evidence
2004	Regulation (EC) No 726/2004 laying down Community procedures for the authorisation and supervision of medicinal products for human and veterinary use and establishing a European Medicines Agency	Governance + practices (regulation)
2006	Regulation (EC) No 1831/2003 of the European Parliament and of the Council of 22 September 2003 on additives for use in animal nutrition: banned the use of antibiotics as growth promoters	Practices (ban)
2010	EMA launches the ESVAC project to develop a harmonised approach for the collection and reporting of data on the use of antimicrobial agents in animals from EU and European Economic Area Member States	Evidence
2011	Action plan against the rising threats from AMR in humans and animals. Renewal of the 2001 commitment (COM (2001) 333 final). One Health approach is established (expired in 2016, and renewed in 2017). Decree/communication/law: COM(2011) 748	Governance
	Joint programming initiative on AMR (JPIAMR) set up by the EU to better coordinate and align worldwide AMR research efforts	Governance
2013	Commission Implementing Decision of 12 November 2013 on the monitoring and reporting of antimicrobial resistance in zoonotic and commensal bacteria (Decision 2013/652/EU)	Evidence
	New Drugs 4 Bad Bugs (ND4BB) project with EUR 700 million of budget for antibiotic development. This project is part of the innovative medicines initiative, a EU public–private partnership funding health research and innovation	Practices (research/innovation)
2015	Commission notice – guidelines for the prudent use of antimicrobials in veterinary medicine (2015/C 299/04)	Practices (guidelines)
	Animal health regulation, Regulation (EU) 2016/429 on transmissible animal diseases and amending and repealing certain acts in the area of animal health (‘Animal Health Law’). In effect from 21 April 2021. It focuses on better prevention and control of listed animal diseases and introduces various measures in general and provides a legal basis for the harmonised monitoring of animal pathogens	Practices (regulation)
2017	EU One Health action plan against AMR: COM(2017) 0339	Governance
	European Union Joint Action on Antimicrobial Resistance and Healthcare-Associated Infections (EU-JAMRAI)	Governance
	Regulation (EU) 2017/625 on official controls to ensure the application of food and feed law, rules on animal health and welfare, plant health and plant protection products	Practices (control)
2019	Regulation (EU) 2019/6 on veterinary medicinal products. In effect from 28 January 2022	Practices (regulation)
	Regulation (EU) 2019/4 on the manufacture, placing on the market and use of medicated feed. In effect from 28 July 2022	Practices (regulation)
	Commission communication – strategic approach to pharmaceuticals in the environment (COM(2019) 128)	Governance
2020	A pharmaceutical strategy for Europe	Governance
	Farm-to-fork strategy: objective of reducing by 50 % the overall EU sales of antimicrobials for farmed animals and in aquaculture by 2030 (COM(2020) 381)	Governance
	Commission Implementing Decision (EU) 2020/1729 on the monitoring and reporting of antimicrobial resistance in zoonotic and commensal bacteria	Evidence
2021	Procedure for the creation of a new EU authority, named the Health Emergency Preparedness and Response Authority (HERA)	Governance
	EU4Health programme 2021–2027	Governance
2022	Entry into force of new regulations on veterinary drugs and medicated feed	Practices (regulation)

In 1986, Sweden was the first country in the world to introduce a legislation to ban the use of AMs as AGPs. In 1995, with the EU's enlargement to include Sweden, the pressures for more prudent use of antibiotics in European farms intensified. In 1995, Denmark, Germany and Norway raised concern that the use of AGP avoparcin in animal feed could encourage resistance to certain AMs used in human medicine. On this basis, avoparcin was banned in all EU Member States in April 1997. Finally, in 2003, the regulation on feed additives phased out the use of AMs as AGPs in the EU, by setting a total ban from 2006, 20 years after Sweden. This decision was significantly influenced by the scientific opinion issued in 1999 by the European Commission's Scientific Steering Committee about the prevalence and development of AMR and its implications for human and animal health.

Since 1998, the European AMR Surveillance Network (EARS-Net), now run by the European Centre for Disease Prevention and Control (ECDC), has been collecting information from all EU Member States regarding invasive bacteria isolated from blood and cerebrospinal fluid in hospitalised patients.

During 2000–2009, potential hazards from AMR originating from AMU in farms were under growing attention, and they were finally included in the 2011–2016 European action plan against the rising threats from AMR, as well as in the 2017 One Health action plan against AMR. The 2017 action plan builds on three main pillars:

• making the EU a best practice region by improving evidence, coordination and surveillance, and control measures on AMU and AMR;
• boosting research, development and innovation by filling current knowledge gaps, providing novel solutions and tools to prevent and treat infectious diseases, and improving diagnosis to control AMR spread;
• intensifying EU efforts worldwide to shape the global agenda on AMR and related risks.

Since the implementation of the 2017 One Health action plan, important updates have been made to further strengthen the EU response to AMR, as detailed in Table 2. Leading intergovernmental organisations and the EU have launched policy strategies that target more prudent use of AMs in both human and veterinary medicine, as well as in the environment, to face the rising threat of AMR. This includes more attentive monitoring of the use of AMs in humans and animals, the spread of infections from resistant bacteria, and the presence of resistant zoonotic and commensal bacteria in animal farms and along the food supply chain. To raise awareness about this issue, the ECDC founded the European Antimicrobial Awareness Day, which aims to provide a platform and support for national campaigns about prudent AMU. Over the years, European Antimicrobial Awareness Day – marked annually in November together with the World Antimicrobial Awareness Week organised by WHO – has developed into a platform of global reach, partnering up with many countries outside the EU as well as with relevant stakeholders, in line with the Commission's One Health approach to AMR.

To promote harmonised monitoring of AMR in zoonotic and commensal bacteria in the food chain, in 2013 the European Commission appointed the European Medicines Agency (EMA) as the lead agency in the collection of data on sales of veterinary AM agents in the Member States. EMA consults with stakeholders, including the ECDC, the European Food Safety Authority (EFSA) and the European Community Reference Laboratory for Antimicrobial Resistance (EURL-AMR). The legislation fosters comparability between the Member States and between the human and veterinary sectors, and facilitates the monitoring of patterns of multidrug resistance. In addition, the ESVAC project collects information on how AM medicines are consumed in animals across the EU [4]. Individual EU Member States provide examples of effective policy implementation to monitor AMU and AMU benchmarking systems for farmers and veterinarians to reduce the overall use of AMs in food production (e.g. yellow card in Denmark, mandatory targets in the Netherlands), which have not yet been upscaled to the EU level.

The EU system for the monitoring of zoonoses was established in 1992. It was improved through better data comparability and monitoring of additional zoonoses, of AMR (e.g. in *Salmonella* and *Campylobacter*) and of food-borne outbreaks in 2003. The first report based on such data was published by EFSA in 2004. In 2013, detailed rules were set for the harmonised monitoring and reporting by the Member States of AMR in several bacteria obtained from samples from certain food-producing animal populations. EFSA and the ECDC jointly published ‘The European Union summary report on antimicrobial resistance in zoonotic and indicator bacteria from humans, animals and food’. In 2020, the European Commission proposed to lay down new technical requirements for AMR monitoring and reporting. They address known implementation issues while scientifically responding to the constantly evolving threat of AMR and ensuring continuity in assessing future trends in AMR after 2020 https://eur-lex.europa.eu/legal-content/EN/TXT/?uri=uriserv:OJ.L_.2020.387.01.0008.01.ENG.

Since the creation of the Quadripartite we've seen an increase in actions and concerns about the issues of AMR in livestock production at the global level. However, it is important to notice that such concerns date back to the 1960s and 1970s in individual EU Member States and subsequently by the Union (the EU common institutions). Some countries were/are in the vanguard of these actions, such as Denmark, Norway, Sweden and the United Kingdom, for instance, and drove the common actions at the Union level during the late 1980s and in the 1990s and 2000s. Measures have been gradually introduced in all the EU Member States. Further restrictions, guidelines and good practices are being progressively introduced as well. Monitoring institutes and methods are also being standardised, and further budget for individual and common actions is being made available.

## 4 Factors affecting antimicrobial use in European Union Member States

Table 3 reports the results of the SYS-GMM estimation. As expected, the AM consumption is positively correlated with the lagged AM consumption, which reflects the patterns of previous consumptions of AMs in the countries. A higher share of pigs in livestock production also leads to a greater consumption of antibiotics, indicating that the production of this species must receive additional attention and support to reduce the AM consumption.

**Table 3:** Regression analysis for panel data with SYS-GMM.

LNMGPCU	Coefficient	Standard error	*Z*	*P > Z*	95 % confidence interval	
LN_MGPCU						
L1.	0.876376	0.044642	19.63	0.000⁎⁎⁎	0.788879	0.963873
Onehealth	-0.03829	0.019694	-1.94	0.052*	-0.07689	0.000313
NAP_Prog	-0.0011	0.020974	-0.05	0.958	-0.0422	0.040012
VET_edu	-0.04155	0.024421	-1.7	0.089*	-0.08941	0.00632
VET_serv	-0.04069	0.020269	-2.01	0.045⁎⁎	-0.08041	-0.00096
AMU_mon	0.032746	0.093078	0.35	0.725	-0.14968	0.215175
AMR_surv	0.034967	0.023738	1.47	0.141	-0.01156	0.081492
BIOSEC_prac	0.035168	0.024425	1.44	0.15	-0.0127	0.08304
POULTRY	0.293592	0.259227	1.13	0.257	-0.21448	0.801667
PIGS	0.36345	0.202043	1.8	0.072*	-0.03255	0.759447
Year						
2015	0	(empty)				
2016	0.525596	0.239486	2.19	0.028⁎⁎	0.056212	0.994981
2017	0.534219	0.244063	2.19	0.029⁎⁎	0.055865	1.012573
2018	0.540242	0.247032	2.19	0.029⁎⁎	0.056069	1.024415
2019	0	(omitted)				
2020	0.593352	0.238204	2.49	0.013⁎⁎	0.12648	1.060224
2021	0.525133	0.232788	2.26	0.024⁎⁎	0.068878	0.981388
2022	0.364101	0.237379	1.53	0.125	-0.10115	0.829355
_CONS	0	(omitted)				
Observations	156					

⁎⁎⁎ p < 0.01,

⁎⁎ p < 0.05,

⁎ p < 0.1;

Improving governance through the implementation and enhancement of multisector and One Health collaboration/coordination leads to substantial reduction in AM consumption. Access to more information about AMU and improved surveillance system for AMR in animals are not significantly affecting AMU. Enhancing training and professional education on AMR in the veterinary sector has a positive impact on the reduction of AMU. Finally, improvements in the performance of the veterinary sector, have also contributed to a reduction in AMU.

As the model includes lagged variables, the first year of data (2015) is not be used in the regression, as the lagged terms are be missing. The year 2019 is omitted, and hence it is our reference year. The intercept is also omitted because it becomes redundant when we include a full set of year dummies. The year dummies collectively capture the constant term, with each year dummy capturing the deviation of that particular year from the reference year (2019). Thus, in terms of interpreting our coefficients, the effects are relative to the year 2019.

## 5 Discussion

The overview of policies and strategies presented in Section 3 shows that legislation in European countries has been very concerned with limiting the veterinary use of critically important AMs to situations where they are the last resort. In some countries, certain critically important AMs (e.g. third-, fourth- and fifth-generation cephalosporins, fluoroquinolones, macrolides, colistin) have been limited to culture-proven infections, or have been subject to special taxation. By 2022, several new EU regulations on veterinary medicinal products had been introduced in national legislation. The implementation of Regulation (EU) 2019/6 is the most important in this respect, as this regulation sets out rules regarding the placing on the market, manufacturing, import, export, supply, distribution, pharmacovigilance, control and use of veterinary medicinal products. As a result of the implementation of this regulation, countries will have to expand their monitoring efforts, as they will also have to monitor the use of antifungals, antiprotozoals, antivirals and topical AMs at livestock farms. The implementation of this regulation also means that, in addition to data on the amount of AMs used in food-producing animal species, data will also have to be collected about other animals that are bred or kept, including animals kept in sectors other than food production (e.g. companion animals).

**Fig. 1:**
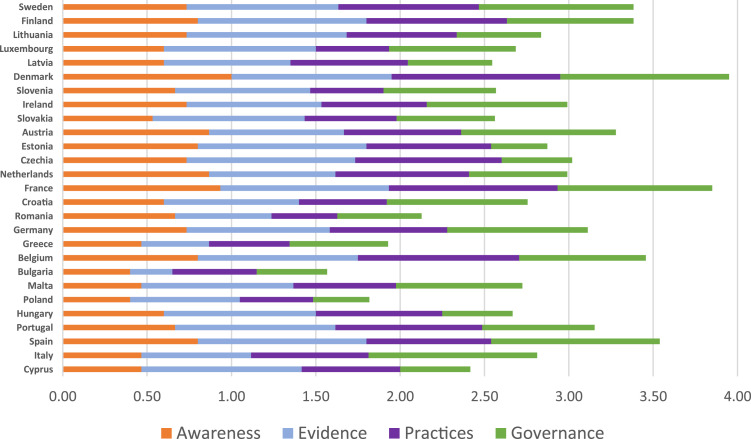
Member States’ comparison of policies and measures adopted in the four domains of awareness, evidence, practices and governance related to AMU and AMR in food and animals, according to the self-assessement in the 2023 TrACSS. Countries are ordered from the lowest to the highest AM consumer in mg/pcu according to the ESVAC data 2018.

The objective of the EU policy is not to phase out AMs from animal production but to guide farmers to more cautious utilisation, by reducing prophylactic and metaphylactic treatments as much as possible and controlling more tightly the prescription, marketing, storage and administration of these medicines, especially the active principles that are of critical importance for human health. To achieve this partial decoupling of livestock farming from AMU, the EU is deploying a mix of measures that include (i) new stricter regulations on AMU and traceability, (ii) the monitoring of AMU and presence of AM-resistant microorganisms in farms, environment and the supply chain, (iii) awareness campaigns addressed to farmers, other stakeholders and the general public, (iv) incentives for the improvement of biosecurity, alternative treatments in farms and the development of private standards related to AMU in livestock production, (v) training for farmers and veterinarians, (vi) finance to scientific research and (vii) coordinated initiatives at the global level. The current situation of AM policies at the level of the EU Member States is, however, quite inhomogeneous: in some countries the national legislation already complies with the new EU measures and is even stricter in some aspects, while other countries have missed the 2022 implementation deadline. Fig. 1 presents a comparison of the stage of development of the NAPs among the EU Member States, where the maximum achievable score is 4 (i.e. 1 in each category). Here, an overview and comparison of the progress achieved so far by EU Member States in their AMU/AMR policies and strategies is presented. Fig. 1 also helps to visualise which measures have been taken by the countries in the vanguard that can support countries that present poor performances in terms of high AMU. In the supplementary materials we included details on how the scores were elaborated.

The countries that obtained the highest total scores identify the weak points of their NAPs implementation in the categories ‘awareness’, more specifically in training and education on AMR for farmers and supply chain operators, and ‘governance’, to fully involve in the plan all relevant sectors (i.e. animal health, food processing and safety) with defined monitoring and evaluation processes in place, compared with the other categories. Practices of biosecurity and good animal husbandry to reduce the use of antimicrobials and minimize development and transmission of AMR in terrestrial animal production also show low scores. The same holds for the adoption of AWaRe classification of antibiotics in the National Essential Medicines List. Most of the countries examined indicated that they do not use AMU and AMR monitoring data from all relevant sectors to amend their national strategies and inform decision-making. As total scoring decreases, the policy areas most affected by insufficient development/implementation are ‘awareness’ and ‘practices’, followed by ‘governance’. In general, ‘evidence’, especially regarding the AMU monitoring capacity, is the policy area where the countries examined are evaluated as having the best levels of NAP implementation with respect to the global action plan's standards.

The results from some important livestock-producing countries show the possibility of overcoming the trade-off between reduced AMU and production performances. The achievements in reducing AMU are a result of several main contributing factors, including long experience of evidence-based guideline implementation, strong participatory local commitment, and integration between actions at the local and national levels; in short, strengthened governance. Improved awareness and training about AMU and AMR for farmers, but especially for veterinarians, who are generally the farmers’ immediate advisors on these topics, can have a quick and strong positive impact on AMU and AMR. Countries must also invest in increased knowledge through their monitoring systems to identify the hotspots, sectors, farmers and regions that should be prioritised for increasing the cost–benefit efficiency of measures.

Additionally, the findings from the quantitative analysis indicate a positive correlation between current and previous patterns of AMU, emphasising the need to address past consumption habits. They also highlight the impact of livestock production, particularly a higher proportion of pigs, on antibiotic consumption, suggesting the importance of targeted interventions in this sector. Improving governance through NAPs and promoting collaboration among different sectors via the One Health approach have been shown to significantly reduce AM consumption [[37], [38], [39]]. Improvements in the performance of the veterinary sector, achieved through the implementation of a plan to address capacity gaps in compliance with WOAH standards on Veterinary Services quality, have also contributed to a reduction in AMU. Last, enhancing training and education on AMR in the veterinary sector positively affects the reduction of AM usage. These findings provide valuable insights for developing effective strategies to combat AMR [21].

Overall, our results show that countries’ AMU/AMR policies are at different stages of progress, and these figures reveal the difficulties faced by countries in properly fighting AMR as a common global threat, with national and international policies and strategies. The scale of the challenge is therefore clear, as is the multitude of areas for policy improvement to achieve satisfactory results at the global scale. It requires global (One Health) governance for health, calling into action stakeholders directly involved in AMU/AMR. Individual levels of commitment and perceptions of the problem, but also intricacies related to the different interest groups, organisations and infrastructures, increase the complexity of managing the AMR problem at a global scale [40].

## 6 Concluding remarks

This study examines the policy strategies aimed at addressing AMU and AMR in animal farms and the food supply chain within EU Member States. It does so by reviewing both current and past policies on the subject. Additionally, it incorporates a quantitative approach to assess the impact of certain policies on reducing AMU in livestock production. The European Union's approach to regulating antimicrobial usage (AMU) in veterinary medicine is multifaceted, encompassing tighter regulations, increased surveillance of AMU, public awareness campaigns, incentives for biosecurity improvements, education and training initiatives for farmers and vets, and financial support for scientific research. However, there is considerable variation in how these policies have been implemented across EU member states. Our results show that countries with a lower AMU score tend to have underdeveloped policies in the areas of 'awareness' and 'practices', while those with higher scores often identify weaknesses in 'awareness', particularly in relation to farmer and supply chain operator education, and 'governance'. Despite this, most countries demonstrated strong capacity in 'evidence', particularly in terms of AMU monitoring.

The study also highlighted the role of veterinarians in advising farmers on AMU and AMR, pointing to the need for increased training and education in this area. Furthermore, the analysis found a correlation between current and past AMU patterns, indicating that efforts to reduce AMU should also address historical consumption habits. Enhancing governance in countries through the implementation and refinement of multisector and One Health collaboration/coordination has also been demonstrated to reduce AMU. The complex relationship between AMR and food safety necessitates a holistic approach to public health. The focus on the pig sector is critical, given its high AMU, underscoring the need for sector-specific interventions, careful antimicrobial stewardship, and enhanced biosecurity measures to effectively mitigate AMR risks and ensure the safety of the food chain.

The EU's approach to AMU regulation reflects a 'One Health' perspective, recognizing the interconnectedness of human, animal, and environmental health. However, the varied progress in implementing AMU/AMR policies across EU member states underscores the complexity of addressing AMR as a global threat. Achieving effective global governance for health requires the active involvement of all stakeholders in AMU/AMR, while also acknowledging the intricacies of different interest groups, organizations, and infrastructures. The challenge is significant, but the potential for policy improvement is vast, offering hope for achieving meaningful results on a global scale.

## CRediT authorship contribution statement

**Caetano Luiz Beber:** Writing – review & editing, Writing – original draft, Visualization, Validation, Software, Project administration, Methodology, Investigation, Formal analysis, Data curation, Conceptualization. **Maurizio Aragrande:** Writing – review & editing, Supervision, Funding acquisition, Conceptualization. **Massimo Canali:** Writing – review & editing, Supervision, Resources, Project administration, Funding acquisition, Conceptualization.

## Appendix Supplementary materials

Image, application 1
