# Supplementary material for: Policies and strategies to control antimicrobial resistance in livestock production: A comparative analysis of national action plans in European Union Member States
Source: Health Policy. 2025 Feb;152:105238. doi: 10.1016/j.healthpol.2024.105238 (PMC11797552; doi:10.1016/j.healthpol.2024.105238)
Supplement: Supplementary file 1 [file mmc1.docx]

Equation 1:

$${MgPCU}_{i,t} = \alpha+ {\beta MgPCU}_{i, t - 1} + \gamma\boldsymbol{X}_{i,t} + \delta\boldsymbol{Z}_{i,t} + \theta_{t} +v_{i} + \varepsilon_{it},$$

**Supplementary material - TrACSS questions**

A comparison of countries’ NAPs on AMR, using the FAO report on *Monitoring Global Progress on Addressing Antimicrobial Resistance* [22], data from the Global Database for the Quadripartite Antimicrobial Resistance (AMR) and the [Country Self-assessment Survey (TrACSS)](http://amrcountryprogress.org/). This information is used to illustrate the measures to fight AMR in these four policy domains, with indicators self-assessed by those countries and their respective progress levels.

For each of the policy domains, a broad set of actions might be implemented or not in a country or sector, in a progressive manner. They can range from being very preliminary or basic to very developed, multisectoral and covering the whole country. In the TrACSS, the countries’ responses may be A to E (where A indicates 1 and E indicates 5), or ‘Yes’ or ‘No’, depending on the question. To compare countries, we assigned scores on a scale from 1 to 5 (Where A = 1 and E = 5, No = 0, and Yes = 1), based on the responses and described below. For ex.: In the 'awareness' category, the sum of all three indicators amounts to a total of 15 points. If a country accumulates 10 points, its resultant score would be 0.67. It is important to clarify that we have consciously decided not to assign varying weights to different categories. This is primarily because we wish to avoid making subjective judgments about the relative importance of each indicator within any given category. We believe that all indicators within a category hold equal relevance, and hence, should carry the same weight.

The scores were totalled for 22 parameters, which report the countries’ answers to the main questions related to food and animal production and questions on actions associated with awareness, evidence, practices and governance. Others, related to humans, plants and the environment, were not considered. Although simplistic, such scoring gives an idea of comparability among countries and to some extent reflects their progress related to AMU and AMR control in livestock production.

### Awareness on NAPs: How the countries evaluated themselves

1. **Raising awareness and understanding of AMR risks and response**:

E - Targeted, nationwide government-supported activities regularly implemented to change behavior of key stakeholders within sectors, with monitoring undertaken over the last 2-5 years = 5 points

D - Nationwide, government-supported antimicrobial resistance awareness campaign targeting all or the majority of priority stakeholder groups, based on stakeholder analysis, utilizing targeted messaging accordingly within sectors = 4 points

C - Limited or small-scale antimicrobial resistance awareness campaign targeting some but not all relevant stakeholders = 3 points

B - Some activities in parts of the country to raise awareness about risks of antimicrobial resistance and actions that can be taken to address it = 2 points

A - No significant awareness-raising activities on relevant aspects of risks of antimicrobial resistance = 0 points

1. **Training and professional education on AMR in the veterinary sector:**

E - AMR is systematically and formally incorporated in curricula for graduating veterinarians and veterinary paraprofessionals and continuing professional training is a formal requirement = 5 points

D - Continuing professional training on antimicrobial resistance and antimicrobial use is available nationwide for veterinary related professionals = 4 points

C - AMR and prudent use of antimicrobial agents are covered in core curricula for graduating veterinarians and for veterinary paraprofessionals in some educational institutions = 3 points

B - Ad hoc AMR training courses available for veterinary related professional = 2 points

A - No training of veterinary related professionals (veterinarians and veterinary paraprofessionals) related to AMR = 0 points

1. **Training and professional education on AMR in farming sector (animal and plant), food production, food safety and the environment:**

E - Tailored AMR training courses are routinely available nationwide and completion of training is a formal requirement for all key stakeholders = 5 points

D - Tailored AMR training courses are routinely available nationwide for all key stakeholders and completion of training is a formal requirement for at least two groups of key stakeholders = 4 points

C - Tailored ad hoc AMR training courses are available for all or the majority of key stakeholders = 3 points

B - Tailored ad hoc AMR training courses available for at least two groups of key stakeholders = 2 points

A - No training provision on AMR for key stakeholders, e.g. farmers and farm workers, extension workers, food and feed processors and retailers, environmental specialists = 1 point

### Evidence on NAPs: How the countries evaluated themselves

1. **Do you have a national plan or system in place for monitoring sales/use of antimicrobials in animals?**

Yes = 1 point; No = 0

1. **The national surveillance system for antimicrobial resistance (AMR) in animals (terrestrial and aquatic):**

E - National system of AMR surveillance established for priority animal pathogens, zoonotic and commensal bacterial isolates which follows quality assurance processes in line with intergovernmental standards. Laboratories that report for AMR surveillance follow quality assurance processes = 5 points

D - Priority pathogenic/ commensal bacterial species have been identified for surveillance. Data systematically collected and reported on levels of resistance in at least one of those bacterial species, involving a laboratory that follows quality management processes, e.g. proficiency testing = 4 points

C - Some AMR data is collected locally but a standardized approach is not used. National coordination and/or quality management is lacking = 3 points

B - National plan for AMR surveillance in place but capacity (including laboratory and for reporting data on AMR) is lacking = 2 points

A - No national plan for a system of surveillance of AMR = 0 points

1. **AMR surveillance is routinely undertaken in animals for the following categories: [Animal (terrestrial and/or aquatic) isolates linked to animal disease.]** Yes = 1 point; No = 0
2. **AMR surveillance is routinely undertaken in animals for the following categories: [Zoonotic pathogenic bacteria]** Yes = 1 point; No = 0
3. **AMR surveillance is routinely undertaken in animals for the following categories: [Commensal isolates]** Yes = 1 point; No = 0
4. **AMR surveillance is routinely undertaken in animals for the following categories: [Specific resistance phenotypes such as ESBL producing indicator E.coli obtained from healthy animals in key food producing species]** Yes = 1 point; No = 0

**National AMR Laboratory network in animal health and food safety sectors (questions 10 and 11):**

1. **Effective integration of laboratories in the AMR surveillance**

E - All laboratories performing AST are integrated in the national AMR surveillance system, have a clear position, and are linked to a national network coordinated by a National Reference Laboratory = 5 points

D - All laboratories performing AST are integrated in the AMR surveillance system but the role should be better formalized and the network better and developed = 4 points

C - Some laboratories performing AST are integrated in the national AMR surveillance system= 3 points

B - Laboratories perform antimicrobial susceptibility testing (AST) for own purposes and are not included in the national AMR surveillance system = 2 points

A - Information not available = 0 points

1. **Level of the standardization and harmonization of procedures among laboratories included in the AMR surveillance system:**

E - 100% of laboratories use the same Antimicrobial Susceptibility Test (AST) guidelines = 5 points

D - Between 80% and 99% of laboratories follow the same AST guidelines = 4 points

C - Between 30% to 79% of laboratories follow the same AST guidelines = 3 points

B - No standardized national AST guidelines are in place or less than 30% laboratories follow the same AST guidelines = 2 points

A - Information not available = 0 points

### Practices on NAPs: How the countries evaluated themselves

1. **Country has laws or regulations on prescription and sale of antimicrobials for animal use**: Yes = 3 points; No = 0
2. **Progress with strengthening veterinary services**

E - Documented evidence of strong capacity in compliance with OIE standards on the quality of Veterinary Services = 5 points

D - Monitoring of Veterinary Services performance carried out regularly, e.g. through PVS Evaluation Follow Up missions = 4 points

C - Implementation of plan to strengthen capacity gaps in Veterinary Services underway = 3 points

B - Veterinary services assessed and plans developed to improve capacity, through a structured approach such as OIE Performance of Veterinary Services (PVS) Evaluation and PVS Gap Analysis missions = 2 points

A - No systematic approach at national level to strengthening Veterinary Services = 0 points

1. **The country has laws or regulations that prohibits the use of antimicrobials for growth promotion in the absence of risk analysis**: Yes = 1 points; No = 0
2. **Biosecurity* and good animal husbandry practices* to reduce the use of antimicrobials and minimize development and transmission of AMR in terrestrial animal production:**

E - Implementation of the nation-wide plan is monitored periodically = 5 points

D - Nationwide implementation of plan to ensure good production practices and national guidance published and disseminated = 4 points

C - National plan agreed to ensure good production practices in line with international standards (e.g. OIE Terrestrial and Aquatic Codes, Codex Alimentarius). Nationally agreed guidance for good production practices developed, adapted for implementation at local farm and food production level = 3 points

B - Some activities in place to develop and promote good production practices: Switzerland. From outside, India is also in this group = 2 points

A - No systematic efforts to improve good production practices = 0 points

1. **Adoption of AWaRe classification of antibiotics in the National Essential Medicines List**

E - Country has incorporated AWaRe classification of antibiotics into its antimicrobial stewardship strategies = 5 points

D - Country is monitoring its antibiotic consumption based on the AWaRe classification of antibiotics = 4 points

C - Country has adopted the AWaRe classification of antibiotics in their National Essential Medicines List = 3 points

B - Country has knowledge about the AWaRe classification of antibiotics and country has intention to adopt it in the next few years = 2 points

A - Country has no knowledge or information about the AWaRe classification of antibiotics = 0 points

1. **Optimizing antimicrobial use in animal health (terrestrial and aquatic):**

E - Enforcement processes and control are in place to ensure compliance with legislation = 5 points

D - The national regulatory framework for AM products incorporates all the elements included in the related international standards on responsible and prudent use of antimicrobials (e.g. OIE Terrestrial and Aquatic Codes, Codex Alimentarius) according to animal species and/or production sector = 4 points

C - National legislation covers all aspects of national manufacture, import, marketing authorization, control of safety, quality and efficacy and distribution of antimicrobial products = 3 points

B - National legislation covers some aspects of national manufacture, import, marketing authorization, control of safety, quality and efficacy and distribution of antimicrobial products = 2 points

A - No national policy or legislation regarding the quality, safety and efficacy of antimicrobial products, and their distribution, sale or use = 0 points

1. **Has a national assessment of risks for residues of antimicrobial compounds and antimicrobial resistant pathogens in the environment been conducted?** Yes = 1 point; No = 0

###

### Governance on NAPs: How the countries evaluated themselves

1. **Multi-sector and One Health collaboration/coordination**:

E - Integrated approaches used to implement the national AMR action plan with relevant data and lessons learned from all sectors used to adapt implementation of the action plan = 5 points

D - Joint working on issues including agreement on common objectives = 4 points

C - Multi-sectoral working group(s) is (are) functional, with clear terms of reference, regular meetings, and funding for working group(s) with activities and reporting/accountability arrangements defined = 3 points

B - Multi-sectoral working group(s) or coordination committee on AMR established with Government leadership = 2 points

A - No formal multi-sectoral governance or coordination mechanism on AMR exists = 0 points

1. **Sector actively involved in developing and implementing the AMR National Action Plan- [Animal Health (terrestrial and aquatic)]** Yes = 1 point; No = 0
2. **Country progress with the development of a NAP on AMR:**

E - National AMR action plan has funding sources identified, is being implemented, and has relevant sectors involved with a defined monitoring and evaluation process in place = 5 points

D - National AMR action plan approved by government that reflects Global Action Plan objectives, with a budgeted operational plan and monitoring arrangements = 4 points

C - National AMR action plan developed = 3 points

B - National AMR action plan under development = 2 points

A - No national AMR action plan = 0 points

1. **Is the country using relevant antimicrobial consumption/use and/or antimicrobial resistance data to amend national strategy and/or inform decision making, at least annually [Animal health (terrestrial and aquatic)]** Yes = 1 point; No = 0
